# Mechanism and Potential Target of Blood-Activating Chinese Botanical Drugs Combined With Anti-Platelet Drugs: Prevention and Treatment of Atherosclerotic Cardiovascular Diseases

**DOI:** 10.3389/fphar.2022.811422

**Published:** 2022-06-03

**Authors:** Dan Li, Yujuan Li, Shengjie Yang, Zongliang Yu, Yanwei Xing, Min Wu

**Affiliations:** ^1^ Guang’an Men Hospital, China Academy of Chinese Medical Sciences, Beijing, China; ^2^ Xiyuan Hospital, China Academy of Chinese Medical Sciences, Beijing, China

**Keywords:** atherosclerosis, target, mechanism, atherosclerotic cardiovascular diseases, blood-activating Chinese botanical drugs, anti-platelet drugs

## Abstract

Atherosclerotic cardiovascular diseases (ASCVDs) are the most important diseases that endanger people’s health, leading to high morbidity and mortality worldwide. In addition, various thrombotic events secondary to cardiovascular and cerebrovascular diseases need must be considered seriously. Therefore, the development of novel anti-platelet drugs with high efficiency, and fewer adverse effects has become a research focus for preventing of cardiovascular diseases (CVDs). Blood-activation and stasis-removal from circulation have been widely considered as principles for treating syndromes related to CVDs. Blood-activating Chinese (BAC botanical drugs, as members of traditional Chinese medicine (TCM), have shown to improve hemodynamics and hemorheology, and inhibit thrombosis and atherosclerosis. Modern medical research has identified that a combination of BAC botanical drugs and anti-platelet drugs, such as aspirin or clopidogrel, not only enhances the anti-platelet effects, but also reduces the risk of bleeding and protects the vascular endothelium. The anti-platelet mechanism of Blood-activating Chinese (BAC) botanical drugs and their compounds is not clear; therefore, their potential targets need to be explored. With the continuous development of bioinformatics and “omics” technology, some unconventional applications of BAC botanical drugs have been discovered. In this review, we will focus on the related targets and signaling pathways of anti-atherosclerotic treatments involving a combination of BAC botanical drugs and anti-platelet drugs reported in recent years.

## Introduction

ASCVDs are the leading cause of morbidity worldwide, especially in some developed countries (Rosenblit, 2019). Platelet aggregation, adhesion, and activation lead to thrombus formation and are associated with CVDs (Showkathali and Natarajan, 2012). Anti-platelet drugs, such as aspirin, are the cornerstone for the treatment and prevention of cardiovascular and cerebrovascular diseases and other thrombotic diseases (Desborough and Keeling, 2017). However, previous studies reported that anti-platelet drugs can inhibit normal haemostasis, increased the risk of bleeding (Yuhara et al., 2014). In the context of these adverse reactions, blood-activating Chinese botanical drugs combined with anti-platelet drugs may become a powerful strategy and treatment for ASCVDs.

The common TCM syndrome of cardiovascular and cerebrovascular diseases is blood stasis syndrome, and its pathological mechanism may involve hypercoagulation, activated platelet aggregation, and abnormal haemorheology (Li et al., 2020). Blood-activation and stasis-removal from circulation has been widely considered as a principle for treatment of syndromes related to CVDs. Moreover, blood-activating Chinese (BAC) botanical drugs have many positive properties, such as multi-target and multi-component therapeutic activities, and mild side effects (Liu et al., 2012). As a member of TCM, BAC botanical drugs have been produced by standardized industrial procedures and are used for treating patients with cardiovascular and cerebrovascular diseases (Liu et al., 2019). There is a growing body of clinical trials that has suggested that the treatment of anti-platelet drugs combined with BAC botanical drugs demonstrated an unexpected effect, however, the relevant targets and mechanisms were still not clear, and the assessments of bleeding risk and adverse reactions need further investigation. The continuous development of bioinformatics technology and proteomics has facilitated the study of anti-platelet therapy combined with Chinese and Western medicine. This review focused on the mechanisms and potential targets of the anti-atherosclerotic effects of BAC botanical drugs combined with anti-platelet drugs.

## Ethnopharmacological Context of Blood-Activating Chinese Botanical Drugs and Research Status

TCM is being manufactured as drugs containing ingredients of standardized quality and quantity. Studies have shown that TCM has demonstrated a positive impact on thrombotic diseases *via* multiple pathways (Kim and Park, 2019). Recently, the contributions of medicinal plants, especially BAC botanical drugs, are getting popularized for their anti-platelet effects. Several studies have revealed that plant extracts including coumarins, xanthones, alkaloids, flavonoids, anthraquinones, and stilbenes may possess the anti-platelet and fibrinolytic activities (Mekhfi et al., 2004; Saluk-Juszczak et al., 2010; Seo et al., 2012; Yoo et al., 2014). Modern applications of BAC botanical drugs in general healthcare are mainly focused on blood stasis syndrome-related diseases such as CVDs for their definite anti-platelet effects (Liu et al., 2012). On the one hand, most of BAC botanical drugs possess the characters of inhibiting the platelet aggregation, for example, Xiongshao Capsule (two grains each time, tid) (Lu et al., 2006), Tongxingluo Capsule (two grains each time, tid) (Wang et al., 2004) can reduce the platelet aggregation rate (PAR). Further, BAC botanical drugs can also inhibit the platelet release reaction. Glycoprotein Ib (GPIb) or integrin αIIbβ3, Van Villebrand factor (vWF) and fibrinogen are the guarantee of platelet adhesion and aggregation (Vajen et al., 2017). Platelets can release different storage granules (α-particles, dense particles, and lysosomes) and secreted products, including clotting factors, growth factors, chemokines, cytokines, prostaglandins and thromboxane A2 (TXA2), which affect many physiological and pathophysiological processes other than hemostasis (Liu et al., 2020). Previous studies suggested that Xue Fu Zhu Yu decoction (40 mg/ml or 80 mg/ml) can inhibit the adenosine diphosphate (ADP)-induced expression of GPIIb/IIIa compound significantly, which may alle*via*te the extent of coronary heart disease (Li et al., 1999). Moreover, Danhong injection (5 mg Tanshinone, phenolic acid, safflor yellow pigment, and 50 mg flavone) combined with aspirin (100 mg/d) and clopidogrel (75 mg/d) can reduce the expression of CD62p and inhibit platelet activation (Chen et al., 2009).

**FIGURE 1:**
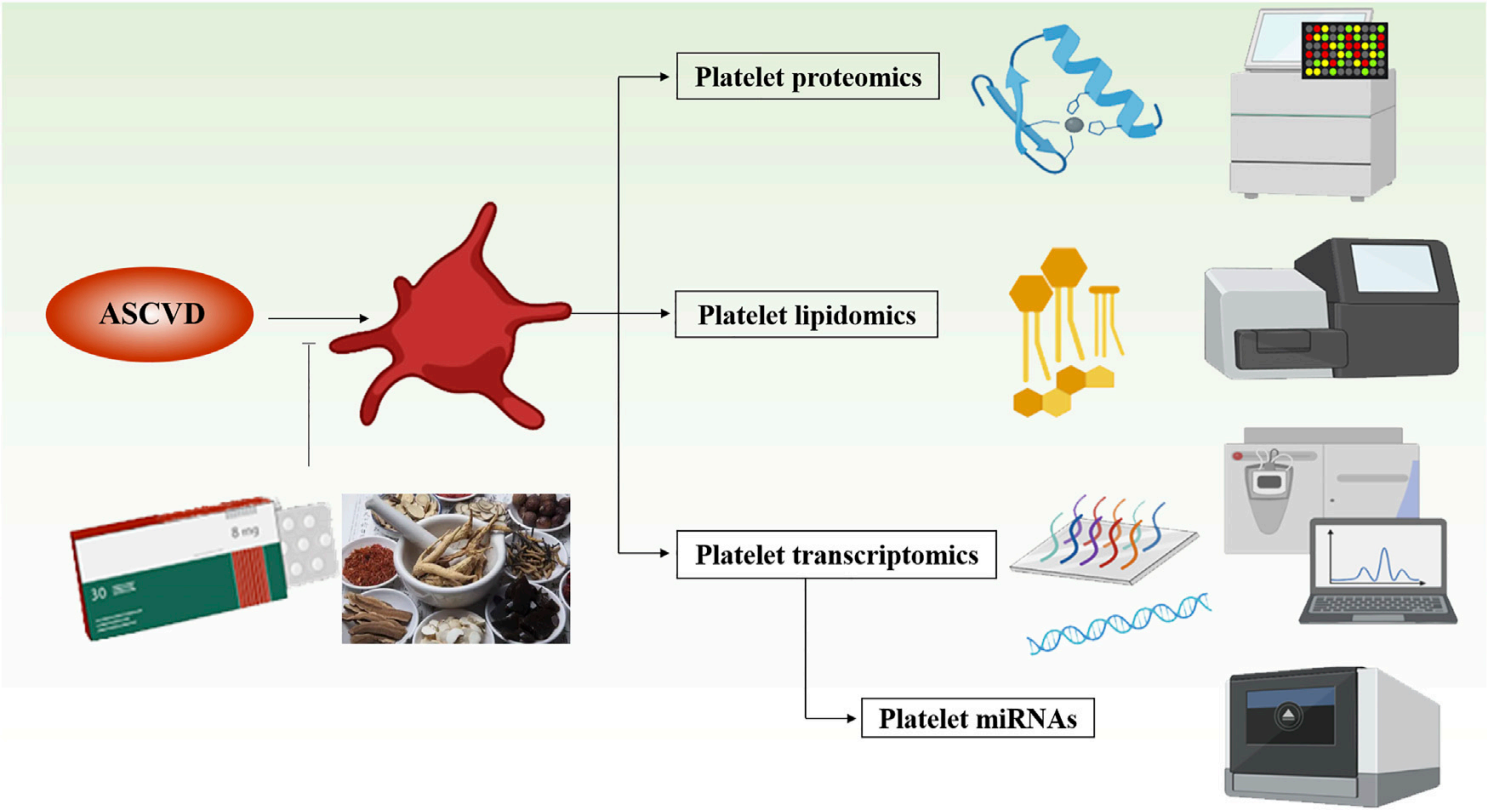
Schematic status on platelet-related omics in the treatment of atherosclerotic cardiovascular disease. This figure was created using BioRender.com.

Apart from these traditional utilizations of these botanical drugs, other mechanisms and targets need our further investigation based on the modern medical technology. At present, more and more studies have highlighted that the platelets can already be used as biomarkers of vascular diseases (Barrett et al., 2020). Some platelet indicators have direct or potential prognostic and diagnostic utility, including platelet count (Vinholt et al., 2016), platelet RNA content (Best et al., 2018), and platelet receptor shedding (Chatterjee and Gawaz, 2017), as well as the levels of circulating platelet monocyte aggregates (Allen et al., 2019) and platelet-derived micro vesicles (Chiva-Blanch et al., 2019). In addition, some “omics” analysis approaches also provide more methods to describe platelets as biomarkers and targets for diagnosis and treatment of diseases (Figure 1). The current research on the platelet transcriptomics (Gutmann et al., 2020), platelet lipidome (Chatterjee, 2020), and proteomics (Dittrich et al., 2008) highlights the changes in microRNAs (miRNAs) expression, disease conditions, and activation states in different individuals and certain specific platelets, which are closely related with the mechanisms of BAC botanical drugs combined with anti-platelet drugs in the prevention and treatment of ASCVDs.

## Mechanism of Blood-Activating Chinese Botanical Drugs Combined With Anti-Platelet Drugs in ASCVDs

### Platelet Proteomics Changes Related to Atherosclerosis

**FIGURE 2:**
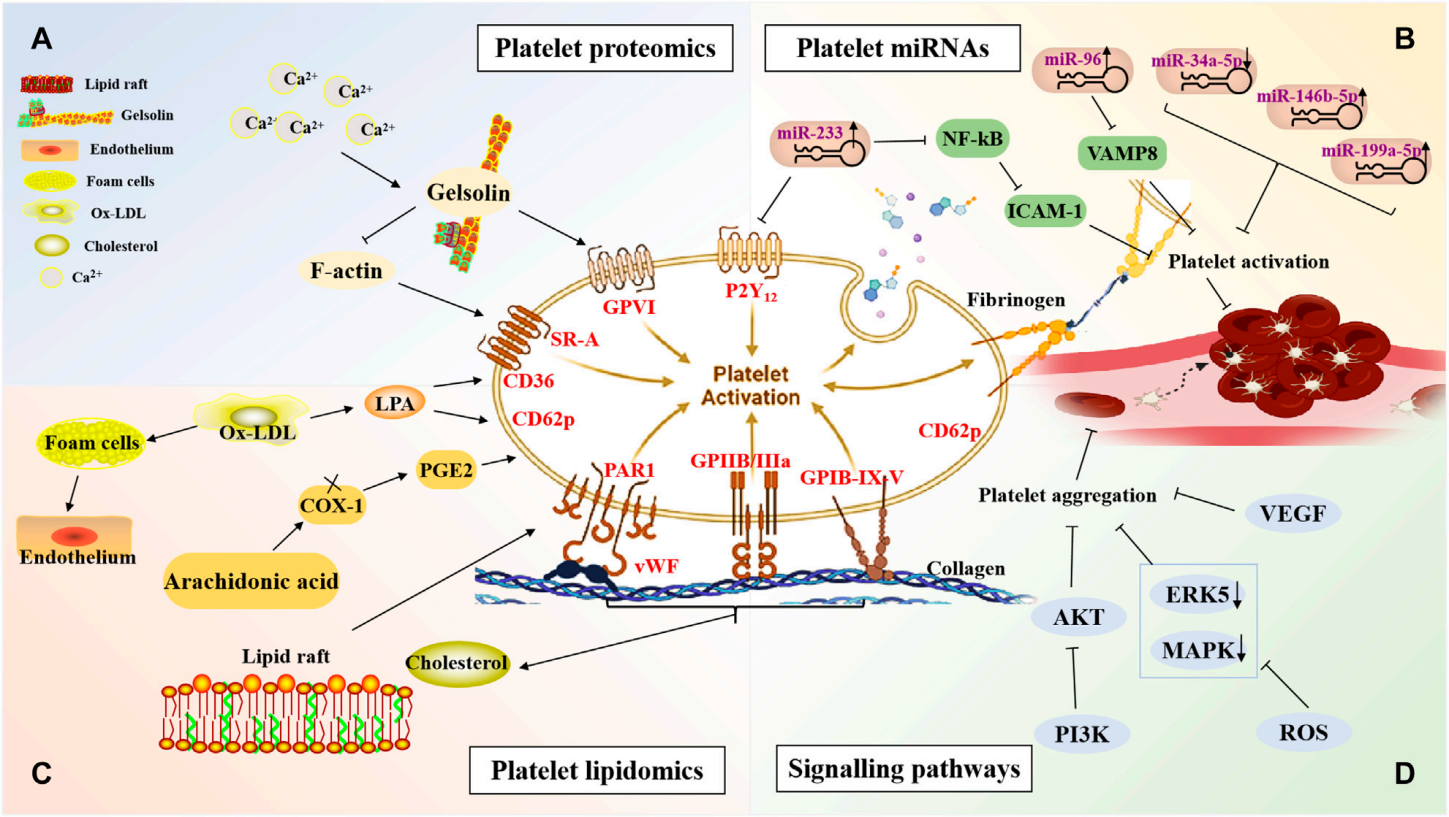
The mechanisms against atherosclerotic cardiovascular diseases of blood-activating Chinese botanical drugs combined with anti-platelet drugs *via* platelet-related targets. This figure was created using BioRender.com. **(A)** The mechanisms of BAC botanical drugs combined with antiplatelet drugs identified through platelet proteomics. **(B)** The mechanisms of BAC botanical drugs combined with antiplatelet drugs involving platelet microRNAs. **(C)** The mechanisms of BAC botanical drugs combined with antiplatelet drugs identified through platelet lipidomics. **(D)** The mechanisms of BAC botanical drugs combined with antiplatelet drugs involving signalling pathways. PAR1, platelet aggregation rate 1; vWF, von Willebrand Factor; P2Y12 reaction units; VAMP8, vesicle-associated membrane protein 8; VCAM-1, vascular cell adhesion molecule-1; COX-1, cyclooxygenase-1; ICAM-1, intercellular adhesion molecule 1; LPA, lysophosphatidic acid; PGE2, prostaglandin E2; SR-A, scavenger receptor A; GPVI, glycoprotein VI; NF-κB, nuclear transcription factor-κB; VEGF, vascular endothlial growth factor; ERK5, Extracellular signal-regulated protein kinase 5; MAPK, mitogen-activated protein kinases; PI3K, Phosphatidylinositol 3-kinase; ROS, reactive oxygen species; ox-LDL, Oxidised low-density lipoprotein.

Platelets are small enucleated cytoplasmic fragments that are derived from mature megakaryocytes in the bone marrow, and their functions, such as activation, adhesion, aggregation, and release of inflammatory factors, are closely related to the dynamic differences in their protein expression or post-translational protein modifications. Therefore, studying the biological properties of platelet proteins using proteomic techniques to identify new platelet signaling proteins, receptors, and target proteins for anti-platelet therapy has become an area of intense research interest (García, 2016). Gelsolin is an important differentially expressed protein in platelet proteomics (Li GH. et al., 2009; Liu et al., 2011; Gupta et al., 2019). Gelsolin interacts with actin to reorganise the cytoskeleton, which is closely associated with platelet activation, inflammatory responses, and cell signal transduction. In addition, platelet gelsolin secretion can be activated when intracellular Ca^2+^ concentrations increase. Gelsolin protein levels are closely related to the formation of blood stasis syndrome in CHD and may be a potential molecular target of BAC botanical drugs against platelet activation and thrombosis. Platelet gelsolin content was significantly higher in patients with CHD with blood stasis syndrome than that in patients without blood stasis syndrome (Li X. F. et al., 2009). Li et al. (Li X. F. et al., 2009) reported that platelet gelsolin levels increased in patients with CHD blood stasis syndrome, which may be attributed to plasma gelsolin depletion. Parguina et al. (Parguiña et al., 2010) found that the platelets of patients with non-ST-segment elevation acute coronary syndrome (non-ST-elevation ACS, NSTE-ACS) differentially expressed proteins, while some of these proteins were involved in platelet activation through the integrin glycoprotein VI (GPVI) signaling pathway (Figure 2A).

### Platelet miRNAs Related to Atherosclerosis

As a class of endogenous small molecular RNAs with post-transcriptional regulatory activity, miRNAs regulate the expression of multiple genes by binding to the 3′ untranslated regions of target mRNAs, thus participating in the pathological process of a variety of diseases (Dangwal and Thum, 2013). miRNAs play an important role in the development of atherosclerosis by regulating the inflammatory response of the vascular wall, lipid metabolism, unstable plaque rupture, platelet activation, thrombosis, and other processes. Platelets contain a variety of mRNA transcripts and miRNAs that regulate mRNA function, suggesting that miRNAs can regulate platelet mRNA expression (Nagalla et al., 2011; Plé et al., 2012; Willeit et al., 2013). Li et al. (2017b) found that the overexpression of miR-223 can inhibit the expression of tissue factors and the pro-coagulation function of endothelial cells (ECs) and can participate in the regulation of thrombosis. Studies have shown that miR-223 derived from platelet exosomes can inhibit the expression of intercellular adhesion molecule 1 (ICAM-1) in coronary ECs by downregulating the NF-κB pathway to regulate thrombosis and inflammatory responses (Landry et al., 2009). Moreover, Laffont et al. (2013) detected P2Y_12_ mRNA in Argonaute 2 (AGO2) immunoprecipitates of platelets, confirming that P2Y_12_ mRNA is a target gene of miR-223, which may affect platelet function and thrombosis by regulating the expression of the P2Y_12_ gene. Shi et al. (Peng et al., 2017; Shi et al., 2013) suggested that the expression of miR-223 in platelets was downregulated in patients with CHD, who were highly responsive to the anti-platelet drug clopidogrel (Campagnolo et al., 2005; Nikolic et al., 2010; Zhang et al., 2014). In addition, miR-96 decreases platelet activity by inhibiting vesicle-associated membrane protein 8 (VAMP8) expression (Shiffman et al., 2008). Studies have shown that miRNAs are not only an important objective reference for the diagnosis of blood stasis syndrome, but they are also important targets of BAC botanical drugs. Compared with that in healthy adults and patients without blood stasis syndrome, the expression of 25 miRNAs in patients with stable angina pectoris (SAP) with blood stasis syndrome was changed, and miR-146b-5p and miR-199a-5p were found to be upregulated (Wang and Yu, 2013a). Studies suggested that the pharmacological effects of ginseng total saponins extract from *Panax ginseng* C. A. Meyer (Araliaceae; *Ginseng Radix et Rhizoma*), such as GS-Rg1, GS-Rb1, and GS-Rh2, can be mediated by miRNAs and their target genes (Figure 2B) (Huang et al., 2019; Wang et al., 2019; Chen et al., 2021).

### Platelet Lipidomics Related to Atherosclerosis

Lipids, as active small molecule metabolites, can enhance the pro-inflammatory and pro-thrombotic effects of platelets (Zhu et al., 2019). They exist in platelets either *via* endogenous synthesis or exogenous uptake and are widely involved in the formation of platelet structures and the regulation of platelet activation signaling pathways (O'Donnell et al., 2014). Oxidised low-density lipoprotein (ox-LDL) not only directly promotes the formation of atherosclerotic plaques, but also promotes platelet activation and thrombosis. For example, the contact between ox-LDL and platelets can promote the formation of foam cells and the activation and deformation of ECs, thus further promoting the occurrence of CHD and thrombotic diseases (Langer et al., 2010). Ox-LDL contains lysophosphatidic acid (LPA) that can activate platelets *via* LPA receptors (Chandler and Hand, 1961; Liao et al., 1995), LPA can stimulate platelet *α*-granule release to translocate P-selectin to the platelet membrane surface, promote the expression of tissue factor in mononuclear cells, promote fibrin formation (Puccetti et al., 2002), and accelerate the formation of intravascular thrombosis (Calkin et al., 2009), thereby leading to acute myocardial infarction (AMI) (Mehta et al., 2001; Mitka, 2001; Haserück et al., 2004). Maixuekang capsule is a kind of BAC botanical drugs, and prepared from Leech (*Whitmania pigra* Whitman) [Hirudinidae; Hirudo]. Liao et al. (2018) found that serum caspase-3 and plasma LPA levels decreased in patients with ischaemic stroke after being treated with Maixuekang Capsule (three grains each time, tid) combined with aspirin (100 mg/d, qd). In addition to LPA receptors, ox-LDL activates platelets *via* CD36 and scavenger receptor A (SR-A) on the platelet surface (Bültmann et al., 2010; Cerletti et al., 2010). Other studies have shown that high cholesterol levels increase platelet production and activity and promote atherosclerotic development and thrombosis. Cholesterol is an important lipid component of lipid rafts. The receptor GPIB-IX-V complex for platelet vWF and the Gi protein-coupled to ADP receptor P2Y_12_ are located on lipid rafts, which are also involved in platelet activation induced by the thrombin receptor PAR1 (Bodin et al., 2003; Bae et al., 2008) (Figure 2C).

### Platelet-Related Signaling Pathways in Atherosclerosis

The MAPK3 signaling pathway is a serine-threonine protein kinase signaling pathway involved in the regulation of inflammation and other responses. Sal*via*nolate injection was prepared from *Salvia miltiorrhiza* Bunge. [Lamiaceae; *Salviae miltiorrhizae radix et rhizoma*], and included 80% magnesium lithospermate B and 20% rosmarinic acid, rosmarinic acid. Through a biomolecular network, Li et al. (2016) found that MAPK8 and MAPK14 may be important signaling pathways for the treatment of SAP using sal*via*nolate injections combined with aspirin. The prescription for Yiqi Huoxue formula mainly includes (*Astragalus membranaceus* (Fisch.) Bunge [Leguminosae; *Astragali Radix*], *Codonopsis pilosula* (Franch.) Nannf. [Umbelliferae; *Codonopsis Radix*], *Salvia miltiorrhiza Bunge* [Lamiaceae; *Salviae miltiorrhizae radix et rhizoma*], *Ligusticum chuanxiong* Hort. [Umbelliferae; Chuanxiong Rhizoma], *Paeonia lactiflora* Pall. [Ranunculaceae; *Paeoniae Radix Rubra*]). And network pharmacological studies revealed that MAPK1 and MAPK8 might be the main targets of the Yiqi Huoxue formula to promote angiogenesis after MI (Wu et al., 2020). In addition, Guo et al. (2020) found that the MAPK pathway may be an important target pathway for Danhong injection combined with anti-platelet drugs in the treatment of patients with chronic SAP (Chen et al., 2009). Extracellular signal-regulated protein kinase 5 (ERK5) is a member of the MAPK family. ERK5 regulates platelet protein expression after MI. Platelet activation in ERK5-deficient mice is weakened after MI, and the expression of 70-kDa ribosomal protein S6 kinase (P70S6K) and RAC1 decreases, thereby prolonging thrombosis time in these mice. Ligustrazine, namely Tetramethylpyrazine, is a pharmacologically active component isolated from the rhizome of the botanical drug *Ligusticum chuanxiong Hort.* [Umbelliferae; *Chuanxiong Rhizoma*], and has been commonly used in China for the prevention and treatment of CVDs. Zhang et al. (2021) found that the intervention of ligustrazine (40 mg/kg^−1^/d^−1^) in rats by gavage significantly reduced reactive oxygen species levels, inhibited the expression of ERK5, P70S6K, and RAC1, inhibited platelet aggregation, adhesion, and release, and inhibited thrombosis without affecting bleeding (Figure 2D).

VEGF is highly specific; it can induce endothelial cell proliferation, promote the formation of new capillaries, and increase vascular permeability. VEGF is involved in the initiation and progression of atherosclerosis (Vm et al., 2016). In addition, VEGF plays an important role in the establishment of myocardial ischaemic collateral circulation (Safi et al., 1999). For example, the combination of the rhizome of *Ligusticum chuanxiong* Hort. [Umbelliferae; *Chuanxiong Rhizoma*] and *Paeonia lactiflora* Pall. [Ranunculaceae; *Paeoniae Radix Rubra*] can promote the expression of VEGF and basic fibroblast growth factor in the ischaemic region of rats with MI, improve left ventricular ejection fraction, promote the angiogenesis of the ischaemic myocardium, and improve cardiac function (Chen et al., 2012). Trichosanthis Fructus pellets is mainly prepared by the extrat from *Trichosanthes kirilowii* Maxim. [Cucurbitaceae; *Trichosanthis Fructus*] with water, 90% ethanol. Zou et al. (Zou et al., 2019) found that the combination of a pre-treatment with Trichosanthis Fructus pellets (1 g/kg^−1^/d^−1^) and aspirin (0.01 g/kg^−1^/d^−1^) in rats by gavage could inhibit platelet aggregation and improve vascular endothelial function by activating TXA2, and prostaglandin I2 (PGI2), and the downstream biological effectors of cyclooxygenase-2 (COX-2) in the VEGF signaling pathway, while also blocking thrombus formation (Figure 2D).

In addition, studies have suggested that miRNAs, as molecular ‘switches’ that regulate genes (Fichtlscherer et al., 2010; Chen and Zhou, 2011), play an important role in regulating angiogenesis (Ward et al., 2021). As an endothelium-specific miRNA, miR-126 plays an important regulatory role in angiogenesis. Microarray analysis showed that the expression of miR-126 inhibits leukocyte adhesion (De Mazière et al., 2008). Zhen et al.(Zhen et al., 2016) established a rat model of MI and found that the treatment of Huoxue Yiqi decoction (1 g/g^−1^/d^−1^) in rats by gavage decreased the expression of miR-126, inhibited the regulation of VEGF by miR-126, and increased the expression of VEGF, thereby promoting angiogenesis. Other studies have shown that miR-126 can also regulate endothelial cell responses to VEGF (Schmidt et al., 2007).

Type I PI3Ks (PI3Kα, PI3Kβ, and PI3Kγ) are expressed in platelets. PI3Kβ is essential for the formation and stability of GPIIb/IIIa, and PI3K can reduce platelet adhesion (Hirsch et al., 2001). Studies have shown that thrombin stimulates the release of platelet *α*-granules through the PI3K pathway; therefore, the PI3K pathway is involved in thrombin- and collagen-induced platelet activation (Penna et al., 2005; Rex et al., 2009). The main components of *Salvia miltiorrhiza* Bunge [Lamiaceae; *Salviae miltiorrhizae radix et rhizoma*] consisting of SAA, SAB, 3-(3,4-Dihydroxy-phenyl)-2-Hydroxy-propionic acid, protocatechuic acid, and catechin. Sal*via*nolic acid A is a water-soluble component, derived from a botanical drug *Salvia miltiorrhiza Bunge* [Lamiaceae; *Salviae miltiorrhizae radix et rhizoma*], It was prepared by 80% ethanol, elution and extraction. An *in vivo* study has shown that sal*via*nolic acid A inhibits platelet activation and reduces arterial thrombosis by inhibiting PI3K (Huang et al., 2010; Liu et al., 2014). In addition, the analysis of a biomolecular network showed that the MAPK and Akt1 pathways are common signaling pathways of sal*via*nolic acid injection combined with aspirin to prevent platelet aggregation and protect vascular ECs (Li et al., 2016). Ginkgolide B, a natural substance extracted from *Ginkgo biloba* L. (Ginkgoaceae). Ginkgolide B (30 mg/kg^−1^/d^−1^) administered by gavage can reduce atherosclerosis in apolipoprotein E null (ApoE^−/-^) mice and inhibit platelet release by blocking the thrombin and collagen activation of the platelet PI3K/Akt pathway (Jackson et al., 2005) (Figure 2D).

## Research on the Anti-Platelet Effect of BAC Botanical Drugs Combined With Anti-Platelet Drugs in ASCVDs

### Evidence From *in vivo* Experiments

Xuesaitong injection (lyophilized) made of total saponins from *Panax notoginseng* (Burk.) F. H. Chen [Araliaceae; *Notoginseng Radix*], which has been widely used in clinical applications. Studies have found that Xuesaitong injection (lyophilized) (40 mg/kg, i. v.) combined with aspirin (10 mg/kg i. g.) and clopidogrel (7.5 mg/kg, i. g.) can protect rats from middle cerebral artery occlusion/reperfusion injury by inhibiting the NOX2/IL-6/STAT3 pathway (Zhu et al., 2021). PGI2 can inhibit the adhesion of adhesion molecules to endothelial cells and play an antithrombotic role. Danhong injection, which consisting of Tanshinone and Sal*via* miltiorrhiza safflower yellow pigment, Sal*via* miltiorrhiza phenolic acid, safflower phenolic glycosides, and catechol, can significantly reduce the mRNA expression of TXA2 and increase the mRNA expression of PGI2 and 6-keto-PGF1 in hyperlipidaemic rats (Fan et al., 2018). Xiongshao capsules (390 mg/kg/day), which contained paeoniflorin (more than or equal to 28 mg each capsule), ferulate (more than or equal to 3.5 mg each capsule), and total phenolic acid (more than or equal to 34 mg each capsule), combined with aspirin 40 mg/kg/day can significantly reduce the platelet gelsolin levels in MI rats, enhance the activity of the plasma actin scavenging system, and inhibit platelet activation (Liu et al., 2013b). Notoginsengnosides (NG) isolated from the root of *Panax notoginseng* (Burk.) F. H. Chen [Araliaceae; *Notoginseng Radix*], is a popularly used traditional Chinese medicine. Another study showed that the expression of 12 platelet proteins in rats treated with Notoginsengnosides differed from that of normal rats. Therefore, Notoginsengnosides promote the expression of some proteins, while inhibiting the expression of others during the process of anti-platelet aggregation (Yao et al., 2008). Sal*via*nolic acid B is the representative component of phenolic acids derived from the dried root and rhizome of *Salvia miltiorrhiza Bunge* [Lamiaceae; *Salviae miltiorrhizae radix et rhizoma*]. Several studies have identified 20 differentially expressed proteins by comparing two-dimensional electrophoresis profiles of sal*via*nolic acid B and normal rat platelets, including platelet aggregation and blood coagulation-related proteins, cell transmembrane signal transduction-related proteins, and cytoskeletal proteins (Ma et al., 2011). Other studies revealed that miR-208a-3p may be a characteristic miRNA of ACS with blood stasis, and miR-222-3p can be used to evaluate the degree of blood stasis during the syndrome (He et al., 2019). Using high-throughput microarray detection, Gao et al. (2021) found that the expression of miR-34a-5p was upregulated in the plasma and myocardial tissues of rats. Ligustrazine has a preventive effect on the formation of coronary microthrombosis in rats, and it may be attributed to its inhibitory effect on miR-34a-5p expression and NF-кB activation (Figure 2B). Recent studies have shown that a variety of lipid metabolites downstream of the arachidonic acid (AA) pathway play important roles in platelet activation and inhibition, as well as gastric mucosal injury and repair (Whittle et al., 1981; Takeuchi, 2014). *Panax notoginseng* (Burk.) F. H. Chen [Araliaceae; *Notoginseng Radix*] (118.8 mg/kg^−1^/d^−1^) enhanced the anti-platelet effect of aspirin by regulating the platelet AA pathway, cyclooxygenase-1 (COX-1) and cytochrome P450 (CYP) metabolism, and gastric mucosal prostaglandin E2 (PGE2) metabolism, through the establishment of an AMI model in rats (Wang et al., 2004). *Salvia deserta Schang* (Labiatae) is a perennial plant belonging to the Lamiaceae family, and belongs to the same family as *Salvia miltiorrhiza Bunge* [Lamiaceae; *Salviae miltiorrhizae radix et rhizoma*]. For example, *Salvia deserta Schang* (Labiatae) 80 mg/kg in 0.5% saline significantly inhibited ADP-induced platelet aggregation in a New Zealand white rabbit anti-platelet aggregation model (Kasimu et al., 2018). Treatment with Taorenchengqi Tang (0.5 g/kg-1/d-1) and aspirin (5 mg/kg^−1^/d^−1^) reduced the infarct volume by inhibiting platelet activation (Li et al., 2017a).

### Evidence From *in vitro* Experiments

Panax notoginseng triol saponins mainly contained Ginsenoside Rg_1_, ginsenoside Re and notoginsenoside R_1_. The treatment of Panax notoginseng triol saponins (50 mg/kg) combined with aspirin (21 mg/kg) in middle cerebral artery occlusion model rats by gavage can reduce adverse gastrointestinal reactions by regulating the TXA2/PGI2 ratio, and enhance the anti-platelet aggregation and anti-thrombotic ability by reducing the adhesion between platelets and damaged vascular endothelial cells mediated by vWF (Xu et al., 2021). Xueshuantong capsule is developed on a traditional Chinese medicine remedy, with a four-herb formula of *Panax notoginseng* (Burk.) F. H. Chen [Araliaceae; *Notoginseng Radix*], *Astragalus membranaceus* (Fisch.) Bunge [Leguminosae; *Astragali Radix*], *Salvia miltiorrhiza Bunge* [Lamiaceae; *Salviae miltiorrhizae radix et rhizoma*] and *Scrophularia ningpoensis Hemsl.* [Scrophulariaceae; *Scrophulariae Radix*]. Studies have found that Xueshuantong can significantly reduce the expression of CD62p on the platelet membrane and inhibit the adhesion of platelets/leukocytes to injured endothelial cells to prevent and treat thrombosis (Han et al., 2019). Ginkgolide B can inhibit the adhesion of platelets to monocytes and reduce VCAM-1 and Cx43 in tumour necrosis factor. Therefore, Ginkgolide B may have an effect on the treatment of inflammation in atherosclerosis (Zhang et al., 2018c). Tanshinone IIA, which is the main fat-soluble component of *Salvia miltiorrhiza Bunge* [Lamiaceae; *Salviae miltiorrhizae radix et rhizoma*], can inhibit LDL oxidation, monocyte endothelial cell adhesion, macrophage cholesterol accumulation, proinflammatory cytokine expression, and platelet aggregation (Gao et al., 2012). Some studies have shown that tanshinone IIA can inhibit platelet activation and downregulate CD36 and mitogen-activated protein kinase 4/Jun N-terminal kinase 2 (CD36/MKK4/JNK2) signals (Wang et al., 2020). *In vitro* studies have shown that Naoxintong capsule, Naoxintong capsule (NXT), developed from Buyang Huanwu Decoction, is a Chinese Materia Medica standardized product extracted from 16 botanical drugs, and can reduce the adhesion of THP-1 monocytes activated by lipopolysaccharides to human umbilical vein endothelial cells (HUVECs) by inhibiting the expression of adhesion molecules and interleukin-6, while it can also reduce the adhesion of platelets activated by ox-LDL to HUVECs (Li et al., 2018). *In vitro* experiments have shown that paeoniflorin combined with ligustrazine significantly inhibits platelet aggregation and activation and reduces the gelsolin content in activated platelets (Liu et al., 2013a).

### Evidence From Clinical Trials and Safety Assessments

Previous studies have shown that the combination of anti-platelet drugs with TCM single-drug extracts, such as ligustrazine and *Panax notoginseng* (Burk.) F. H. Chen [Araliaceae; *Notoginseng Radix*] (Wang et al., 2021), or their compound preparations such as Xuesaitong injection (Wang et al., 2004), can augment the inhibition of platelet aggregation and prevent blood clot formation. Sal*via*nolic acid A decreased the expression of PAC-1 and CD62p and reduced platelet aggregation induced by ADP and thrombin (Zhou et al., 2020). In addition, studies have shown that hirudin, the main component of Maixuekang (four grains each time, tid), is a highly specific thrombin inhibitor, which inhibits platelet adhesion and aggregation, reduces blood viscosity, reduces inflammatory injury of vascular endothelial function, and improves plaque stability (Ge et al., 2012; Yan et al., 2012; Song et al., 2021). However, these studies are still not sufficiently detailed; thus, it is necessary to explore other potential targets. Moreover, the inhibitory effect of *Panax notoginseng* (Burk.) F. H. Chen [Araliaceae; *Notoginseng Radix*] (240 mg/d) on platelet CD62p (P-selectin) and platelet membrane glycoprotein GPIIB/IIIa were better than that of aspirin (50 mg/d) (Wang et al., 2004). Details on the research on anti-platelet drugs combined with BAC botanical drugs are presented in Table 1.

**TABLE 1:** Research on anti-platelet drugs combined with BAC botanical drugs.

BAC Botanical drugs/scientific name	Chemical composition and dosage of formulas	Main research/model	Mechanism/platelet targets	Efficacy	Safety data/side effect	Anti-platelet drugs	References
Danshen/*Salvia miltiorrhiza Bunge* [Lamiaceae; *Salviae miltiorrhizae radix et rhizoma*]	Sal*via*nolatic acid B (Sal-B), 200 mg/day	Clinical trial: 63 patients with ACS	Phosphodiesterase (PDE) and antagonizing P2Y12 receptor↓; platelet activation↓	PAC-1 positive: T vs. C (47.0 ± 10.0% vs. 52.1 ± 6.2%, *p* < 0.05); CD62P expression: T vs. C (39.5 ± 8.3% vs. 45.0 ± 6.7%, *p* < 0.01)	—	Aspirin and clopidogrel, loading dose:300 mg; maintenance dose:100 mg/d and 75 mg/d	Liu et al. (2014)
*Panax notoginseng* saponins*/*[Araliaceae; *Notoginseng Radix*]	Panax notoginseng saponins, 60 mg/d	Clinical trial: 42 patients with stable CHD complicated with chronic gastritis	The activity of platelet COX-1↓; the production of TXB2, PGD2, PGE2, 11-HETE↓; the downstream oxylipids of AA/COX-1 pathway↓	—	Secretion of gastrin and motilin↑, relieved dyspeptic symptoms	Aspirin, loading dose:300 mg; maintenance dose:100 mg/d	Wang et al. (2021)
Sanqi*/Panax notoginseng* saponins [Araliaceae; *Notoginseng Radix*]	Panax notoginseng saponins, 118.8 mg/kg/d	MI rats	The level 6,15-diketo-13,14-dihydro-prostaglandin (PG)F1α, 13,14-dihydro-15-keto-PGE2 and PGE2↓	—	Aspirin -related gastric injury was mitigated	Aspirin, loading dose: 31.25 mg/kg/d; maintenance dose: 31.25 mg/kg/d	Wang et al. (2021)
Ligustrazine injection/*Ligusticum chuanxiong* Hort. [Umbelliferae; *Chuanxiong Rhizoma*]	Chuanxiongzine, 26.16 mg/kg/d	Rabbit thrombus	AA, ADP, PAF-induced platelet aggregation rate↓	PAR: T vs. C (14.6 ± 2.6% vs. 35.7 ± 2.9%, *p* < 0.01); PAIR: T vs. C (65.5 ± 6.2% vs. 15.4 ± 6.8%, *p* < 0.05)	—	Aspirin and clopidogrel, 5.13 mg/kg/d amd 3.85 mg/kg/d	He et al. (2017)
Leech (*Whitmania pigra Whitman*) [Hirudinidae; *Hirudo*]	Leech (*Whitmania pigra Whitman*) powder, 3 g/d	Clinical trial: 42 patients with acute cerebral infarction	vWF↓; GMP-140↓; endothelial injury↓; platelet activation↓	vWF: T vs. C (150.67 ± 13.00 vs. 191.23 ± 15.67, *p* < 0.01); GMP-140: T vs. C (14.90 ± 3.01 vs. 15.03 ± 2.98, *p* < 0.01)	—	Aspirin, 75 mg/d	Wu et al. (2007)
*Ginkgo biloba* leaf extract [Ginkgoaceae; *Ginkgo biloba* L.]	Ginkgolide B, 0.6 mg/ml	HUVECs were incubated with ginkgolide B and aspirin	TNFα-induced expression of VCAM-1, VE-cadherin, and Cx43↓; platelet and monocyte adhesion↓	—	—	Aspirin, 1 mM	Zhang et al. (2018b)
Xuefu zhuyu pill/-	*Peach kernel water extract, safflor yellow pigment, tangerine peel, and saikosaponin*, 18 g/d	Clinical trial: 57 patients with atherosclerosis	vWF↓; GMP-140↓; ɑ-GMP-140↓	ɑ-GMP-140: T vs. C (601 ± 106 vs. 644 ± 87, *p* < 0.01)	—	Aspirin, 40 mg/d	Li et al. (1998)
Taohongsiwu Decoction/-	BAC botanical drugs extracts with water, TSD alcohol extract, 750 ml L^−1^ 90% ethanol, 2 g/ml/d	Clinical trial: 88 patients with stable CHD who underwent PCI	TXB_2_↓; TX B_2_/6-Keto-PGF1ɑ↑; vWF↓; GMP-140↓	TXB_2_/6-Keto-PGF1ɑ: T vs. C (0.55 ± 0.16 vs. 0.53 ± 0.15, *p* < 0.05); GMP-140: T vs. C (16.14 ± 3.03 vs. 16.51 ± 3.45, *p* < 0.01)	—	Aspirin, 10 ml/kg/d	Han et al. (2010)
Danhong injection/*Salvia miltiorrhiza Bunge* [Lamiaceae; *Salviae miltiorrhizae radix et rhizoma*] and *Safflower* [Asteraceae; *Carthami Flos*]	Tanshinone, phenolic acid, safflor yellow pigment, and flavone, 40 ml/d	Clinical trial: 100 patients with ACS	Platelet activation (CD62p, GPⅡb/Ⅲa, FIB-C) and inflammatory response (hs-CRP)↓	CD62p: T vs. C (6.3 ± 1.6 vs. 8.6 ± 1.8, *p* < 0.01); GPⅡb/Ⅲa: T vs. C (15.6 ± 6.5 vs. 28.5 ± 7.3, *p* < 0.01); FIB-C: T vs. C (3.2 ± 1.4 vs. 4.3 ± 1.5, *p* < 0.01); hs-CRP: T vs. C (13.8 ± 8.4 vs. 18.4 ± 8.2, *p* < 0.01)	—	Aspirin and clopidogrel, loading dose:300 mg; maintenance dose:100 mg/d and 75 mg/d	Chen et al. (2009)
Sulfotanshinone sodium injection/-	Sulfotanshinone sodium, 60 mg/d	Clinical trial:100 patients with UAP	FIB and fibrin DD↓	—	—	Aspirin, loading dose:300 mg; maintenance dose:100 mg/d	Yan et al. (2009)
Compound Danshen Dropping Pills/*Salvia miltiorrhiza Bunge* [Lamiaceae; *Salviae miltiorrhizae radix et rhizoma*]	Water-soluble Danshen, 324 mg/kg^−1^/d^−1^	Arteriovenous bypass model in rats	PT↑; APTT↑; TT↑; FIB↓	APTT: T vs. C (43.85 ± 11.81 vs. 33.77 ± 1.40, *p* < 0.05); PT: T vs. C (18.35 ± 0.35 vs. 17.63 ± 0.70, *p* < 0.05); TT: T vs. C (41.13 ± 10.97 vs. 39.2 ± 6.65, *p* < 0.05); hs-CRP: T vs. C (2.11 ± 1.05 vs. 2.75 ± 0.46, *p* < 0.05)	—	Clopidogrel, 30 mg/kg	Ma et al. (2014)
Shexiang Baoxin pills/-	BAC botanical drugs extracts with water, 90% ethanol, and ethyl acetate, 100 mg^−1^/d^−1^	Clinical trial:131 patients with ACS combined with clopidogrel resistance	Platelet aggregation rate↓; serum level of MMP-2↓	—	—	Clopidogrel, 75 mg/d	Zhang et al. (2016)
Bunchang Naoxintong capsule/-	BAC botanical drugs extracts with water, 90% ethanol, and ethyl acetate, loading dose:3.2 g; maintenance dose:1.6 g^−1^/d^−1^	Clinical trial: 90 patients with CYP2C19*2 polymorphism	percent inhibitions of maximum platelet aggregation and late platelet aggregation↓	Maximal aggregation with 5 μmol/L ADP: T vs. C (27.66 ± 8.62 vs. 37.45 ± 10.27, *p* < 0.05); Late aggregation with 5 μmol/L ADP: T vs. C (18.12 ± 9.75 vs. 27.87 ± 9.50, *p* < 0.05)	MACEs (Sudden cardiac arrest and Readmission due to ACS): T vs. C (31.11% vs. 13.33% ± 6.65, *p* = 0.043, OR = 0.341, 95% CI: 0.117–0.990)	Aspirin and clopidogrel, loading dose:300 mg; maintenance dose:100 mg/d and 75 mg/d	Chen et al. (2014)
Tongxinluo capsules/-	BAC botanical drugs (*Salvia miltiorrhiza Bunge* [Lamiaceae; *Salviae miltiorrhizae radix et rhizoma*], Leech (*Whitmania pigra Whitman*) [Hirudinidae; *Hirudo*], Rosewood Heart Wood [Thymelaeaceae; *Aquilariae Lignum Resinatum*] extracts with water, 90% ethanol, 2.6 g/d	Clinical trial:136 patients with ACS after PCI	PRU and hsCRP levels↓	The prevalence of HPR: T vs. C (15.8% vs. 24.8%, *p* = 0.013)	The composite prevalence of ischemic events did not differ significantly (χ2 = 1.587, *p* = 0.208)	Aspirin and clopidogrel, loading dose:300 mg	Zhang et al. (2018a)
Xuesaitong capsule/-	*Panax notoginseng* saponins*/*[Araliaceae; *Notoginseng Radix*], 240 mg/d	Clinical trial:120 patients with hyperviscosity syndrome	TXB_2_↓; TXB_2_/6-Keto-PGF1ɑ↑; ET↓; CD62P AND CD41↓	TXB_2_: T vs. C (64.92 ± 20.51 vs. 66.98 ± 23.85, *p* < 0.05); TXB_2_/6-Keto-PGF1ɑ: T vs. C (153.19 ± 52.05 vs. 147.57 ± 39.66, *p* < 0.05); ET: T vs. C (71.36 ± 17.47 vs. 78.82 ± 30.62, *p* < 0.05); CD62P: T vs. C (11.22 ± 7.24 vs. 15.99 ± 9.95, *p* < 0.05); CD41: T vs. C (40.61 ± 16.01 vs. 48.91 ± 22.14, *p* < 0.05)	T: none; C: gastrointestinal reactions (2 cases); rashes (3 cases)	Aspirin,50 mg/d	Wang et al. (2004)
Xiongshao capsule/-	*Paeonia lactiflora* Pall. [Ranunculaceae; *Paeoniae Radix Rubra*] and *Ligusticum chuanxiong* Hort. [Umbelliferae; *Chuanxiong Rhizoma*], 390 mg/kg^−1^/d^−1^	Rat model of AMI	gelsolin expression↓; the level of plasma F-actin and MFI of platelet calcium ion↓	—	—	Aspirin, 40 mg/kg/day	Liu et al. (2013b)

HUVECs, human umbilical vein endothelial cells; UAP, unstable angina pectoris; CHD, coronary heart disease; ACS, acute coronary syndrome; PCI, percutaneous coronary intervention; MI, acute myocardial infarction; AMI, acute myocardial infarction; TX, thromboxane; COX, cyclooxygenase; ADP, adenosine diphosphate; PAF, platelet activating factor; PAR, platelet aggregation rate; PAIR, platelet aggregation inhibition rate; vWF, von Willebrand Factor; GMP, platelet membrane protein; AA, arachidonic acid; VCAM-1, vascular cell adhesion molecule-1;FIB-C, fibrinogen C; hs-CRP, high-sensitivity C-reactive protein; PT, prothrombin time; APTT, activated partial thromboplastin time; TT, concentration and thrombin time; ET, endothelin; PRUs, P2Y_12_ reaction units; HPR, high platelet reactivity; MFI, mean fluorescence intensity; MACEs, major adverse cardiovascular events; OR, odds ratio; T, treatment group (BAC, botanical drugs combined anti-platelet drugs); C, contrl group (anti-platelet drugs).

Moreover, BAC botanical drugs play an anti-platelet role by reducing platelet adhesion and inhibiting platelet activation, aggregation, and release (Yoo et al., 2018). A clinical study involving 151 patients that were followed up for 1 year, showed that the rate of serious bleeding in patients treated with Naoxintong capsule (1.6 g/d, tid) combined with aspirin (100 mg/d) was lower than that in patients treated with adjusted-dose warfarin [international normalized ratio 2.0–3.0) (0% vs. 7.9%, OR = 0.921, 95% CI: 0.862–0.984, *p* = 0.028) (Wang et al., 2018). The results of a study on 130 patients with acute coronary syndrome (ACS) after PCI for 12 weeks showed that on the basis of secondary prevention of CHD, patients treated with Danhong injections (20 ml Danhong added in 5% glucose 100 ml/0.9% sodium chloride, 100 ml, gtt, qd, 1x/wk) combined with Naoxintong capsules (4 grains, tid) had better clinical outcomes and no obvious adverse reactions, while the levels of endothelin-1 (ET-1) and vWF decreased (Zhao et al., 2018). Further analyses found that anti-platelet therapy with Xuesaitong capsules for 4 weeks, improved the blood indexes of whole blood viscosity and attenuated peripheral blood miR-199-5p and miR-146b-5p expression (Wang J. et al., 2017). A study of 70 patients with NSTE-MI showed that salvianolic acid, a water-soluble active component of the TCM *Salvia miltiorrhiza* Bunge [Lamiaceae; *Salviae miltiorrhizae radix et rhizoma*], downregulated miR-92a, miR-363, miR-499, miR-30b, miR-454-3p, and miR-93 in peripheral blood mononuclear cells, and upregulated miR-144, miR-451, miR-494, and miR-320 (Zhang et al., 2017). The results of clinical studies are presented in Table 1.

Most studies have shown that BAC botanical drugs combined with anti-platelet drugs can reduce the risk of bleeding and improve anti-platelet drug resistance (Wang et al., 2021). Only a few patients show mild gastrointestinal reactions, nausea, and other side effects, and these adverse events are easy to control or spontaneously control (Wang et al., 2021). Tongxinluo capsule is a medicine mainly consisting of *Salvia miltiorrhiza Bunge* [Lamiaceae; *Salviae miltiorrhizae radix et rhizoma*], Leech (Whitmania pigra Whitman) [Hirudinidae; *Hirudo*], Rosewood Heart Wood [Thymelaeaceae; *Aquilariae Lignum Resinatum*]; *Salviae miltiorrhizae radix et rhizoma*] and *Safflower* [Asteraceae; *Carthami Flos*]. A systematic review of 16 studies showed that Tongxinluo reduced the incidence of restenosis (RR = 0.16, 95% CI: 0.07–0.34), myocardial infarction (MI) (RR = 0.32, 95% CI: 0.16–0.66), and a few adverse events including gastrointestinal reactions and nausea (Mao et al., 2015). Another systematic review showed that among 12 randomised controlled trials (RCTs) and 1,044 patients, Danhong injections combined with basic treatments was more effective than Danshen injections combined with western medicine in improving the nerve injury of patients with cerebral infarction. Three RCT cases included four cases of patients with adverse reactions to drugs, such as skin itching, erubescence, rash fever, and low-grade fever, which resolved without clinical intervention (Wang K. et al., 2017). A systematic review of the clinical efficacy and safety of Ginkgo biloba tablets in the treatment of acute cerebral infarction, involving 10 RCTs and 886 participants, demonstrated neurological function and blood lipid regulation improvements through the use of a combination of conventional western medicine and ginkgo leaf tablets, which were more efficient and incurred a lower incidence of adverse reactions than other treatments. Most of the symptoms were mild nausea, gastrointestinal discomfort, dizziness and headache, and limb fatigue, among others (Meng et al., 2021).

## Other Potential Anti-platelet Targets of Blood-Activating Chinese Botanical Drugs Combined With Anti-Platelet Drugs in ASCVDs

Recently, a variety of potential anti-platelet therapeutic targets have attracted increasing attention. Huoxue Rongluo formula was composed of *Rehmannia glutinosa* Libosch.[Scrophulariaceae; *Rehmanniae Radix*] *Spatholobus suberectus* Dunn [Leguminosae; *Callerya reticulata (Benth.) Schot*], *Photinia serratifolia* (Desfontaines) Kalkman*Scrophularia ningpoensis* Hemsl [Scrophulariaceae; *Scrophulariae Radix*], *Boswellia carterii* Birdw.[Buseraceae; *Olibanum*], *Commiphora myrrha* Engl.[Buseraceae; *Myrrha*] and *Ligusticum chuanxiong* Hort. [Umbelliferae; *Chuanxiong Rhizoma*] in the ratio of 6:6:3:2:3:2:2:2. Using network pharmacology, Yang et al. (2020) found that the Huoxue Rongluo formula mainly promoted angiogenesis in cerebral infarctions by regulating oxidative stress, cell apoptosis, the proliferation of ECs and smooth muscle cells, and the hypoxia-inducible factor 1 (HIF-1) and mechanistic target of rapamycin (mTOR) signaling pathways. Ginkgo biloba B is one of the most active PAF receptor antagonists of known natural products. Ginkgo biloba diterpene lactone me.

Glumine injections, the main component of which is Ginkgo biloba diterpene lactone, have been used in the treatment of cerebral infarction (Yang et al., 2011). Shenwu Guanxin granules (6.48 g/kg), which is a kind of standardized suspension extracted from 17 BAC botanical drugs, can downregulate the expression of Fas and Bax, increase Bcl-2 levels, and decrease the content of caspase-3, while simultaneously regulating the death receptor and mitochondrial pathways, and playing a role in anti-ischaemic reperfusion (I/R) injury and apoptosis of ischaemic cardiomyocytes in rats (Jiang et al., 2006). The combination of *Panax notoginseng* (Burk.) F. H. Chen [Araliaceae; *Notoginseng Radix*] and *Paeonia lactiflora* Pall. [Ranunculaceae; *Paeoniae Radix Rubra*] can significantly improve the haemorheology of rats with blood stasis syndrome, inhibit platelet aggregation, downregulate the expression of Bax, upregulate the expression of Bcl-2, reduce apoptotic signaling to inhibit neuronal apoptosis, and reduce the ischaemic area.

P-selectin is a promising potential target for anti-platelet drug research. Wang et al. (2015) found that *Rabdosia rubescens* (Hemsl.) Hara [Labiatae; Rabdosia rubescens] exerts an anti-platelet aggregation effect by targeting the release of P-selectin in rats. Studies have shown that CHD blood stasis syndrome is positively correlated with the level of CD62p; thus, anti-platelet therapy can be performed by reducing the expression of CD62p (Liu et al., 2011). For example, in a clinical phase I probe trial, Wilson et al. (2018) successfully demonstrated the high oral bioavailability of BMS-986120 in healthy people; however, the risk of thrombosis has not been fully demonstrated. *Salvia miltiorrhiza Bunge* [Lamiaceae; *Salviae miltiorrhizae radix et rhizoma*]*, Safflower* [Asteraceae; *Carthami Flos*], and *Ligusticum chuanxiong* Hort. [Umbelliferae; *Chuanxiong Rhizoma*] have certain calcium antagonistic activities that inhibit platelet aggregation, thereby indicating that free intracellular Ca^2+^ in the platelets is also one of the targets of TCM (Wang et al., 2013b; Li et al., 2019). In addition, several novel anti-platelet molecular-targeted therapies are under development and involved in clinical trials.

## Conclusion

The activation and aggregation of platelets play important roles in the formation of thrombosis and the initiation of cardiovascular events. Therefore, Anti-platelet medications are important anti-atherosclerotic treatments and reduce the occurrence of cardiovascular events. The constituents of TCM are complex. We need to combine BAC herbs with anti-platelet drugs properly to reduce the risk of adverse drug reactions. At present, the exploration of multiple biological functions of platelets based on proteomics, lipidomics, and miRNA has become an area of intense research to clarify the targets and anti-atherosclerotic mechanisms of BAC botanical drugs combined with anti-platelet drugs. The analysis of platelet protein expression profiles of different patients before and after clinical drug intervention can help identify and discover the common targets of BAC botanical drugs combined with anti-platelet drugs and help us further understand the mechanism of action of BAC botanical drugs against atherosclerosis. Most studies have shown that interventions using BAC botanical drugs combined with anti-platelet drugs can affect the expression of miRNAs, which in turn affect downstream genes, proteins, cytokines, and their networks, thus playing important pharmacological roles. Lipids exist in platelets and are involved in the regulation of the platelet activation signaling pathway. Lipid rafts are key to the regulation of platelet receptor function. Therefore, lipids can further enhance pro-inflammatory responses after oxidative modification and metabolism. Furthermore, MAPK, VEGF, and PI3K-Akt signaling pathways are important antiplatelet-related pathways, which play important roles in anti-atherosclerosis.

At present, research on the application of BAC botanical drugs combined with anti-platelet drugs is progressing rapidly. The application of BAC botanical drugs combined with anti-platelet drugs can help prevent the disadvantage of single targeting by modern anti-platelet drugs to a certain extent, while enhancing their anti-platelet activities and without increasing the incidence of thrombotic events. However, there are still some limitations to this research. Proteomics has been successfully used in platelet research. However, it is not clear whether the excessive F-actin released upon tissue injury after platelet activation stimulates the platelets to secrete gelsolin, platelet gelsolin, and if the platelet gelsolin can be therapeutically targeted by the anti-platelet action of Chinese botanical drugs to promote blood circulation and remove blood stasis (Kim and Park, 2019). Active lipid products produced during the process of platelet metabolism play an important role in regulating platelet function and promoting the inflammatory responses. However, the effect of various of lipids is still unclear, and the research on the interaction between lipids and platelets is not sufficiently detailed. The mechanism underlying the interaction of lipids, platelets, and inflammatory mediators in atherosclerosis requires further study. The level of miRNA-specific expression in platelets may be a diagnostic indicator of platelet activation and an important objective reference for the diagnosis of blood stasis syndrome. However, the responses of miRNAs to anti-platelet drugs are not completely consistent. More studies are needed to provide the data about the anti-platelet drug reactivity of miRNAs for clinical samples.

The constituents of TCM are complex, and most clinical studies have small sample size, which is restricting the use of TCM in anti-platelet therapy. The mechanisms of BAC botanical drugs combined with anti-platelet drugs need further exploration. Therefore, it is necessary to conduct a scientific assessment of the efficacy and safety of BAC botanical drugs combined with anti-platelet drugs. In addition, a large number of basic experiments and large-sample, multi-centre clinical studies are required to provide a basis for the application of BAC botanical drugs combined with anti-platelet drugs.
